# Interhemispheric functional connectivity asymmetry is distinctly affected in left and right mesial temporal lobe epilepsy

**DOI:** 10.1002/brb3.2484

**Published:** 2022-02-14

**Authors:** Xu Zhao, Huicong Kang, Zhiqiang Zhou, Ying Hu, Juan Li, Shihui Li, Jia Li, Wenzhen Zhu

**Affiliations:** ^1^ Department of Radiology Tongji Hospital Tongji Medical College Huazhong University of Science and Technology Wuhan China; ^2^ Department of Neurology Tongji Hospital Tongji Medical College Huazhong University of Science and Technology Wuhan China; ^3^ Department of Anesthesiology and Pain Medicine Tongji Hospital Tongji Medical College Huazhong University of Science and Technology Wuhan China

**Keywords:** asymmetry, functional connectivity, hippocampal sclerosis, mesial temporal lobe epilepsy, resting‐state functional connectivity magnetic resonance imaging

## Abstract

**Introduction:**

The differences of functional connectivity (FC) and functional asymmetry between left and right mesial temporal lobe epilepsy with hippocampal sclerosis (LMTLE and RMTLE) have not been completely clarified yet. The purpose of the present study is to investigate the FC changes and the FC asymmetric patterns of MTLE, and to compare the differences in FC and functional asymmetry between LMTLE and RMTLE.

**Methods:**

In total, 12 LMTLE, 11 RMTLE patients, and 23 healthy controls (HC) were included. Region of interest (ROI)‐based analysis was used to evaluate FC. The right functional connectivity (rFC) and left functional connectivity (lFC) of each ROI were calculated. Asymmetry index (AI) was calculated based on the following formula: AI=100×(rFC−lFC)/[(rFC+lFC)/2]. Paired *t*‐test and univariate analysis of variance were used to analyze FC asymmetry. Linear correlation analysis was performed between significant FC changes and lateralized ROIs and epilepsy onset age and duration.

**Results:**

LMTLE and RMTLE patients showed different patterns of alteration in FC and functional asymmetry when compared with controls. RMTLE presented more extensive FC abnormalities than LMTLE. Regions in ipsilateral temporal lobe presented as central regions of abnormalities in both patient groups. In addition, the asymmetric characteristics of FC were reduced in MTLE compared with HC, with even more pronounced reduction for RMTLE group. Meanwhile, ROIs presented FC AI differences among the three groups were mostly involving left temporal lobe (L_hippo, L_amyg, L_TP, L_aMTG, and L_pTFusC). No correlation was found between significant FC changes and lateralized ROIs and epilepsy onset age and duration.

**Conclusion:**

The FC and asymmetric features of MTLE are altered and involve both the temporal lobe and extra‐temporal lobe. Furthermore, the altered FC and asymmetric features were distinctly affected in LMTLE and RMTLE compared to controls.

## Graphical abstract

The abnormal FC and asymmetric features of MTLE are altered and involve both the temporal lobe and extra‐temporal lobe. Furthermore, the altered FC and asymmetric features were distinctly affected LMTLE and RMTLE.

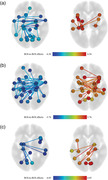

## 1 INTRODUCTION

Mesial temporal lobe epilepsy (MTLE) is one of the most common types of refractory focal epilepsy in adults, and hippocampal sclerosis (HS) is the hallmark of most cases (Cendes et al., 1993). Previous studies have provided evidence that MTLE involved a complex pattern of brain functional changes that are not only confined to temporal lobes but also extended beyond the temporal regions (de Campos et al., 2016; Pereira et al., 2010). Importantly, the functional alterations in MTLE showed asymmetric patterns whereby abnormalities were more obvious in the hemisphere ipsilateral to the epileptogenic focus than the contralateral hemisphere (Pereira et al., 2010; Pittau et al., 2012). Pittau et al. (2012)reported that impairment of functional connectivity (FC) involved both the affected hippocampus and the contralateral “healthy” hippocampus, and the impairment was less severe for the “healthy” hippocampus compared with the affected one. Moreover, some studies showed that MTLE with left HS (LMTLE) presented different functional abnormalities compared with MTLE with right HS (RMTLE) (Besson et al., 2014; de Campos et al., 2016; Li et al., 2015). Li et al. (2015) observed alterations of both sides of the hippocampal network in unilateral MTLE, while RMTLE presented with stronger correlations between intra‐hippocampus FC and disease duration than LMTLE. de Campos et al. (2016) showed that LMTLE was associated with more intricate bilateral dysfunction compared with RMTLE. In addition, LMTLE showed differential effects on cognitive function compared with RMTLE (Doucet et al., 2013; Kim et al., 2003). Verbal memory was mostly affected by LMTLE, whereas visuospatial memory impairment was more prominent in RMTLE (Pereira et al., 2010). The above evidence suggests that HS may have different effects on the pathogenic mechanisms of MTLE depending on whether it is located in left or right hemisphere.

Structural and functional asymmetries have been well documented in the human brain and are thought to be a central principle of nervous system architecture that shapes the functional organization of most cognitive systems (Ocklenburg et al., 2016; Toga & Thompson, 2003). The normal asymmetric pattern in the human brain is beneficial for high‐level functioning, including language, motor abilities, visuospatial attention, face processing, and reasoning (Gotts et al., 2013; Kong et al., 2018). On the contrary, changes in the normal hemispheric lateralization pattern have been associated with many cognitive and neuropsychiatric diseases (Carper et al., 2016; Zhao et al., 2019). For instance, the generally attenuated FC asymmetry in schizophrenia patients increased with disease duration and correlated with psychotic symptoms (Ribolsi et al., 2014). In addition, the altered functional asymmetry of the primary sensorimotor cortex in patients with benign epilepsy and centrotemporal spikes was associated with intelligence quotient scores (Cao et al., 2017). Jung et al. (2018) showed that the FC asymmetry between the amygdala and the intraparietal sulcus was associated with severity of social anxiety symptoms. Furthermore, Gotts et al. (2013) provided direct evidence that functional asymmetry was associated with cognitive ability. Thus, altered functional asymmetry was thought to be correlated with cognitive function in numerous neuropsychiatric diseases.

Due to the importance of HS location in MTLE and the asymmetric characteristics of the human brain, we hypothesized that the differences between LMTLE and RMTLE may be related to the functional asymmetric features of the human brain. Understanding the unique effects of MTLE on FC and functional asymmetries can provide insights into the pathological processes associated with MTLE. However, the exact mechanisms have not been completely clarified. Resting‐state functional connectivity magnetic resonance imaging (rs‐fMRI) is a powerful method to identify plausible FC alterations for the reference region of interest (ROI) in neuropsychiatric disorders, by measuring the correlations of the blood oxygen level dependent (BOLD) signals between different brain regions (Buchbinder, 2016). Therefore, in this study, we used rs‐fMRI to analyze the FC features of both left and right hemispheres as well as the functional asymmetric features of MTLE by directly comparing the FC of the left hemisphere to the right hemisphere. We hypothesized that not only the FC of MTLE but also the functional asymmetries of MTLE would be altered compared with HC, and the FC and functional asymmetries would be distinctly affected in the left and right MTLE.

## 2 METHODS AND MATERIALS

### 2.1 Subjects

We recruited 23 right‐handed adult unilateral MTLE patients diagnosed according to the criteria defined by the Commission on Classification and Terminology of the International League Against Epilepsy (Berg et al., 2010). The patients were then divided into left MTLE (LMTLE) (*n* = 12) or right MTLE (RMTLE) (*n* = 11) groups based on clinical manifestations, video electroencephalography (v‐EEG), neuroimaging results, and/or positron emission tomography‐computed tomography (PET‐CT). To minimize heterogeneity among the patient groups, we excluded MTLE patients with bilateral HS or without HS on MRI. Patients with lesions other than HS, mismatch between seizure semiology, v‐EEG, and neuroimaging results and other neurologic disorders were also excluded. Among these participants, 11 had pathologically confirmed HS by the subsequent surgical treatment, and the other 12 cases of HS were diagnosed according to typical MRI findings (ipsilateral hippocampus atrophy, disturbed internal structure, increased signal intensity on T2FLAIR, and widening of ipsilateral temporal angle) (Malmgren & Thom, 2012). The age of epilepsy onset (first unprovoked seizure) and epilepsy duration were also recorded. In addition, 23 right‐handed, healthy adults with matched age and gender were recruited as controls. There were no lesions on MRI and no self‐reported central nervous system disorders in any controls. Consistency in age and gender among the RMTLE, LMTLE, and HC groups was determined with one‐way analysis of variance (ANOVA) and the χ^2^ test. A Mann–Whitney U test was conducted for the epilepsy onset age and epilepsy duration in the RMTLE and LMTLE groups with the significance level set at *p* < .05. All subjects provided written informed consent and the study was approved by the Institutional Review Board and Medical Ethics Committee of Tongji Hospital, Tongji Medical College of Huazhong University of Science and Technology.

### 2.2 Data acquisition

All subjects were examined by a 3.0‐T MR scanner (Discovery MR750, GE Healthcare, Milwaukee, Wisconsin) with a 32‐channel phased‐array head coil. All subjects underwent standard structural brain scan, including axial T1WI, T2WI, T2FLAIR, and a sagittal three‐dimensional T1‐weighted‐imaging brain volume (3D‐T1BRAVO) (TR/TE/TI 8.2/3.2/450 ms, flip angle 12°, slice thickness 1 mm, sagittal slices 166, matrix size 256 × 256 × 160, FOV 25.6 × 25.6cm^2^, and NEX = 1). In order to better observe the hippocampus and temporal lobe, an oblique coronal T2FLAIR perpendicular to the long axis of the hippocampus was also acquired. Rs‐fMRI data were obtained using a gradient‐recalled echo based on echo planar imaging (GRE‐EPI) sequences with the following acquisition parameters: TR/TE 2000/35 ms, flip angle (FA) = 90°, FOV = 240 mm × 240 mm, matrix = 64 × 64, axial slices = 40, slice thickness = 4 mm with no gap, scan time = 8 min, 240 volumes for each subject. During rs‐fMRI data acquisition, participants were instructed to keep their eyes closed, remain awake, and move as little as possible. Each participant's head was padded with flexible foam to limit head motion.

### 2.3 Data analysis

Preprocessing of the rs‐fMRI data was performed using CONN toolbox version1 (Whitfield‐Gabrieli & Nieto‐Castanon, 2012) (https://www.nitrc.org/projects/conn) running on MATLAB, version R2017a (MathWorks, Inc., Natick, MA, USA). The preprocessing pipeline included the following steps: discard the first 10 volumes to allow for signal stabilization and for participants to acclimatize to scanning noise, realignment, slice‐timing correction, normalization, outlier detection (ART‐based scrubbing), spatial and temporal filtering, and structural segmentation/normalization to Montreal Neurological Institute (MNI) space. To remove unwanted motion, physiological and other artifactual effects from the BOLD signal, a combination of component‐based noise correction (aCompCor) (white matter and cerebrospinal fluid, five components each), scrubbing, motion regression (default 12 regressors), and filtering was used. aCompCor was shown to improve the specificity of functional connectivity estimates (Muschelli et al., 2014). Subsequently, the data were parcellated into 105 cortical and subcortical regions using the FSL Harvard‐Oxford Atlas provided with the toolbox. These ROIs were used as seeds of interest for ROI‐to‐ROI connectivity analyses. The Pearson's correlation coefficient was calculated between the time course of each ROI and the time courses of all other ROIs. For second‐level (between‐subjects) general linear model analyses, the correlation coefficients were converted into normally distributed scores using Fisher's transformation. A height threshold of uncorrected *p *< .01 and an extent threshold of false discovery rate (FDR)‐corrected *p *< .05 at the cluster level were reported.

In addition, the ROIs presented significant abnormalities of FC were included in the next analysis to test hemisphere asymmetry. Five ROIs (Frontal Medial Cortex, Subcallosal Cortex, anterior division of Cingulate Gyrus, posterior division of Cingulate Gyrus, Precuneus Cortex) in the midline involving both hemispheres were excluded. Pearson's correlation coefficients were calculated between the time course of each ROI and the time courses of all residual ROIs in left hemisphere or right hemisphere, which provided a ROI‐to‐ROI connectivity matrix. The correlation coefficients were then converted into normally distributed scores using Fisher's transformation and used for analysis. In order to estimate the FC asymmetry of each ROI in the three groups, we calculated the right FC (rFC) and left FC (lFC) of each ROI, respectively. rFC and lFC are the absolute values of total correlation coefficients strongly correlated (threshold at *r* > 0.25) with the ROI in the right hemisphere and left hemisphere, respectively. We then conducted a paired *t*‐test for the rFC and lFC of each ROI in the HC, LMTLE, and RMTLE groups using SPSS 24. We also calculated the asymmetry index (AI) using the formula AI=100×(rFC−1FC)/[rFC+1FC)/2] (Carper et al., 2016; Galazzo et al., 2016; Reich et al., 2006). A positive AI value indicated that the FC of the ROI in the right hemisphere was greater than the left hemisphere, which was termed rightward asymmetry, while a negative value represented leftward asymmetry. For the statistical comparison of each AI among the three groups, a univariate ANOVA was used, and a post hoc analysis with Bonferroni correction was used for multiple comparisons and to determine the direction of AI differences among groups. Linear correlation analysis was further performed between significant FC and AIs of significant lateralized ROIs and epilepsy age of onset/duration. To correct for multiple comparisons, results were then conducted FDR correction by MATLAB. The FDR‐corrected *p *< .05 results were reported.

## 3 RESULTS

### 3.1 Clinical data

The clinical and demographic characteristics of the three groups are summarized in Table 1 and Table S1. There were no significant differences with regards to gender (*p *= .503) or age (*p *= .051) among the LMTLE, RMTLE, and HC groups. In addition, no significant differences were found in epilepsy duration (*p *= .976) or epilepsy age of onset (*p *= .470) between the LMTLE and RMTLE groups.

**TABLE 1:** Demographic characteristics of the subjects

	LMTLE (*n* = 12)	RMTLE (*n* = 11)	HC (*n* = 23)	Significant differences (*p*‐value)
Age (mean ± SD years)	25.17 ± 3.69	29.25 ± 9.92	31.82 ± 10.25	.051
Gender (male/female)	9/3	6/5	13/10	.503
Age at seizure onset (mean ± SD years)	16.42 ± 8.61	19.09±8.81	NA	.470
Epilepsy duration (mean ± SD years)	12.83 ± 8.68	12.73±6.53	NA	.976

*Abbreviations*: HC, healthy control; LMTLE, left mesial temporal lobe epilepsy with hippocampal sclerosis; NA, not applicable; RMTLE, right mesial temporal lobe epilepsy with hippocampal sclerosis; SD, standard deviation.

### 3.2 Functional connectivity of bilateral hemispheres

#### 3.2.1 Functional connectivity differences between LMTLE and HC

**FIGURE 1:**
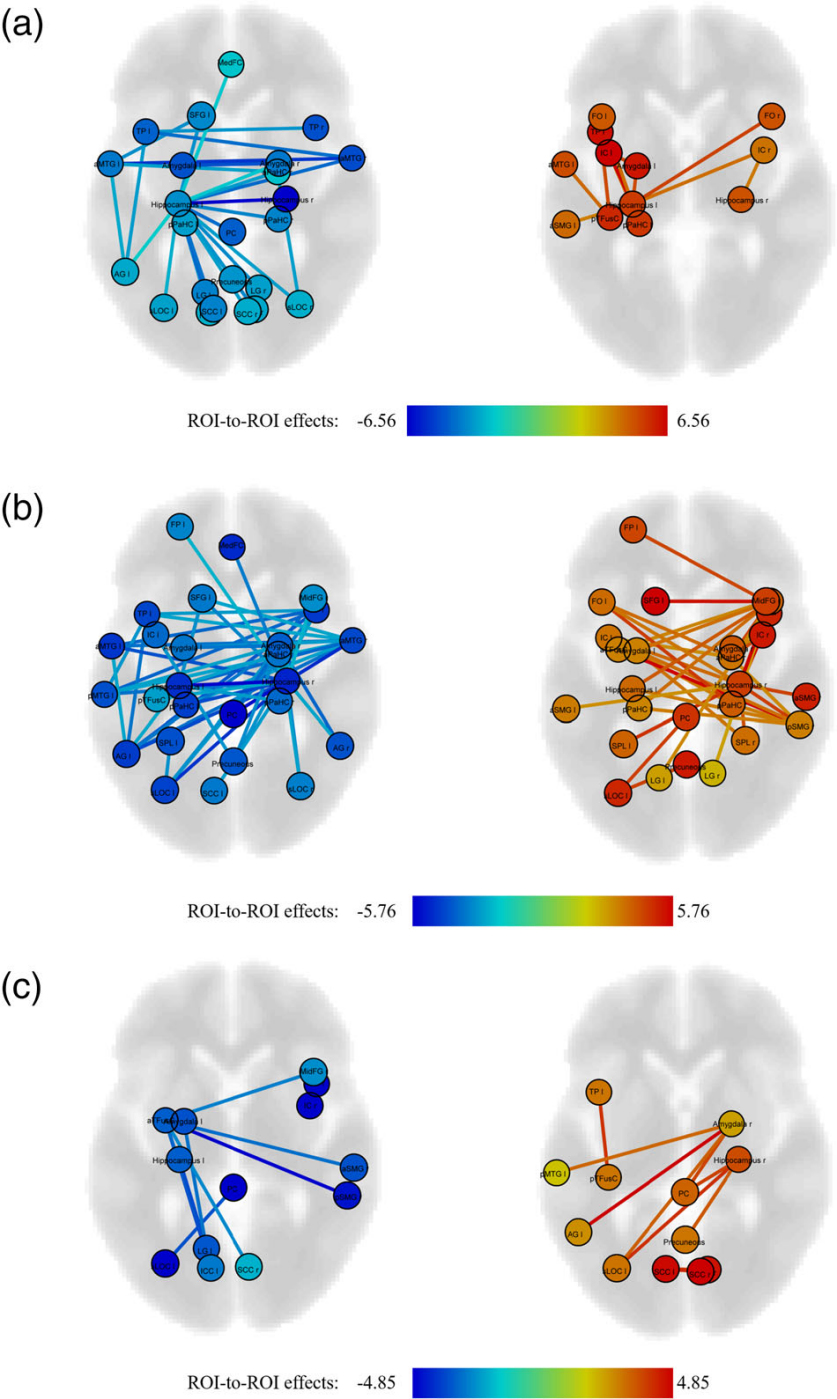
Visualization of different functional connectivity between LMTLE and HC (a), between RMTLE and HC (b), and between LMTLE and RMTLE (c), respectively. Connections are depicted if significant at *P*_FDR_ < 0.05 to represent meaningful findings and trends. Height of the T values is indicated by color as shown in the color bar. AG, angular gyrus; aMTG, anterior division of middle temporal gyrus; aPaHC, anterior division of parahippocampal gyrus; aSMG, anterior division of supramarginal gyrus; aTFusC, anterior division of temporal fusiform cortex; FDR, false discovery rate; FO, frontal operculum cortex; FP, frontal pole; IC, insular cortex; ICC, intracalcarine cortex; LG, lingual gyrus; LMTLE, left mesial temporal lobe epilepsy with hippocampal sclerosis; MedFC, frontal medial cortex, MidFG, middle frontal gyrus; PC, posterior division of cingulate gyrus; pMTG, posterior division of middle temporal gyrus; pPaHC, posterior division of parahippocampal gyrus; pSMG, posterior division of supramarginal gyrus; pTFusC, posterior division of temporal fusiform cortex; RMTLE, right mesial temporal lobe epilepsy with hippocampal sclerosis; ROI, region of interest; SCC, supracalcarine cortex; SFG, superior frontal gyrus; SPL, superior parietal lobule; sLOC, superior division of lateral occipital cortex; TP, temporal pole

In total, there were 39 ROIs of the 105 ROIs (37.14%) showed significant differences of FC among the LMTLE, RMTLE, and HC groups. In particular, there were 30 ROIs (28.57%) showed alterations in LMTLE group when compared to controls. The reduced FC resulted to involve both hemispheres in LMTLE group while the increased FC revealed to be more impaired ipsilateral to the seizure focus when compared to control subjects. Furthermore, the ipsilateral hippocampus and anterior division of middle temporal gyrus (aMTG) stood out as central regions of abnormalities. Specifically, when compared to controls, LMTLE patients showed significantly decreased connectivity mostly between bilateral temporal lobe (bilateral hippocampus [hippo], bilateral aMTG, right anterior and posterior division of parahippocampal gyrus [aPaHC and pPaHC], bilateral temporal pole [TP], and bilateral amygdala [amyg]) and between ipsilateral temporal lobe and bilateral occipital lobes (bilateral lingual gyrus [LG], bilateral supracalcarine cortex [SCC], bilateral intracalcarine cortex [ICC], and bilateral superior division of lateral occipital cortex [sLOC]). In addition, increased FC were observed between regions within ipsilateral temporal lobe, such as the FC of L_hippo‐L_amyg (*P*_FDR_ = 0.004), left posterior division of temporal fusiform cortex (pTFusC)‐L_aMTG (*P*_FDR_ = 0.023), L_pTFusC‐L_TP (*P*_FDR_ = 0.011), and between regions in ipsilateral temporal lobe and bilateral insular cortex (IC) and bilateral frontal operculum cortex (FO) (Figure 1a and Table S2).

#### 3.2.2 Functional connectivity differences between RMTLE and HC

In total, there were 36 ROIs (34.29%) showed alterations in RMTLE group when compared to controls. Both the reduced and increased FC revealed to involve both hemispheres in RMTLE group, and some areas in the ipsilateral temporal lobe stood out as central regions of abnormalities, such as R_hippo, R_amyg, R_aMTG, R_TP, R_aPaHC, and R_pPaHC. Specifically, when compared to controls, RMTLE patients showed significantly decreased connectivity mostly between regions within bilateral temporal lobes, and between ipsilateral temporal lobe and bilateral frontal lobes (R_hippo‐left superior frontal gyrus [L_SFG] [*P*_FDR_ = 0.005], R_hippo‐left frontal pole [L_FP] [*P*_FDR_ = 0.027], R_hippo‐right middle frontal gyrus [R_MidFG] [*P*_FDR_ = 0.027], R_hippo‐frontal medial cortex [MedFC] [*P*_FDR_ = 0.004], R_amyg‐R_MidFG [*P*_FDR_ = 0.006], and R_aMTG‐L_SFG [*P*_FDR_ = 0.030]). Ipsilateral temporal lobe also presented decreased FC with bilateral parietal and occipital lobes (bilateral angular gyrus (AG), L_SCC, bilateral sLOC). In addition, increased FC was observed between regions within bilateral temporal lobes, and between ipsilateral temporal lobe and bilateral FO, bilateral IC, and bilateral superior parietal lobule (SPL). Specifically, the ipsilateral posterior division of supramarginal gyrus (pSMG), IC, and FO were presented as central regions of increased FC as well (Figure 1b and Table S3).

#### 3.2.3 Functional connectivity differences between LMTLE and RMTLE

In total, there were 22 ROIs (20.95%) showed alterations between LMTLE and RMTLE. L_aTFusC presented as a central region of reduced FC in LMTLE when compared to RMTLE. In particular, compared to RMTLE, LMTLE patients showed significantly decreased connectivity between L_aTFusC‐R_aSMG (*P*_FDR_ = 0.031), L_aTFusC‐R_pSMG (*P*_FDR_ = 0.002), L_aTFusC‐R_SCC (*P*_FDR_ = 0.049), L_aTFusC‐R_MidFG (*P*_FDR_ = 0.043), L_aTFusC‐L_ICC (*P*_FDR_ = 0.013), L_aTFusC‐L_LG (*P*_FDR_ = 0.030), L_LG‐L_amyg (*P*_FDR_ = 0.042), L_LG‐L_hippo (*P*_FDR_ = 0.033), R_IC‐R_TP (*P*_FDR_ = 0.017), and L_sLOC‐posterior division of cingulate gyrus (PC) (*P*_FDR_ = 0.039). In addition, R_amyg and R_hippo presented as central regions of increased FC in LMTLE when compared to RMTLE. Specifically, LMTLE patients showed significantly increased connectivity between R_hippo‐precuneus (*P*_FDR_ = 0.028), R_hippo‐PC (*P*_FDR_ = 0.019), R_hippo‐L_sLOC (*P*_FDR_ = 0.019), R_amyg‐PC (*P*_FDR_ = 0.020), R_amyg‐L_AG (*P*_FDR_ = 0.004), R_amyg‐L_sLOC (*P*_FDR_ = 0.030), R_amyg‐L_pMTG (*P*_FDR_ = 0.030), L_SCC‐R_ICC (*P*_FDR_ = 0.015), L_SCC‐R_SCC (*P*_FDR_ = 0.015), and L_pTFusC‐L_TP (*P*_FDR_ = 0.023) than RMTLE patients (Figure 1c and Table S4).

### 3.3 Functional connectivity asymmetry in the three groups

**FIGURE 2:**
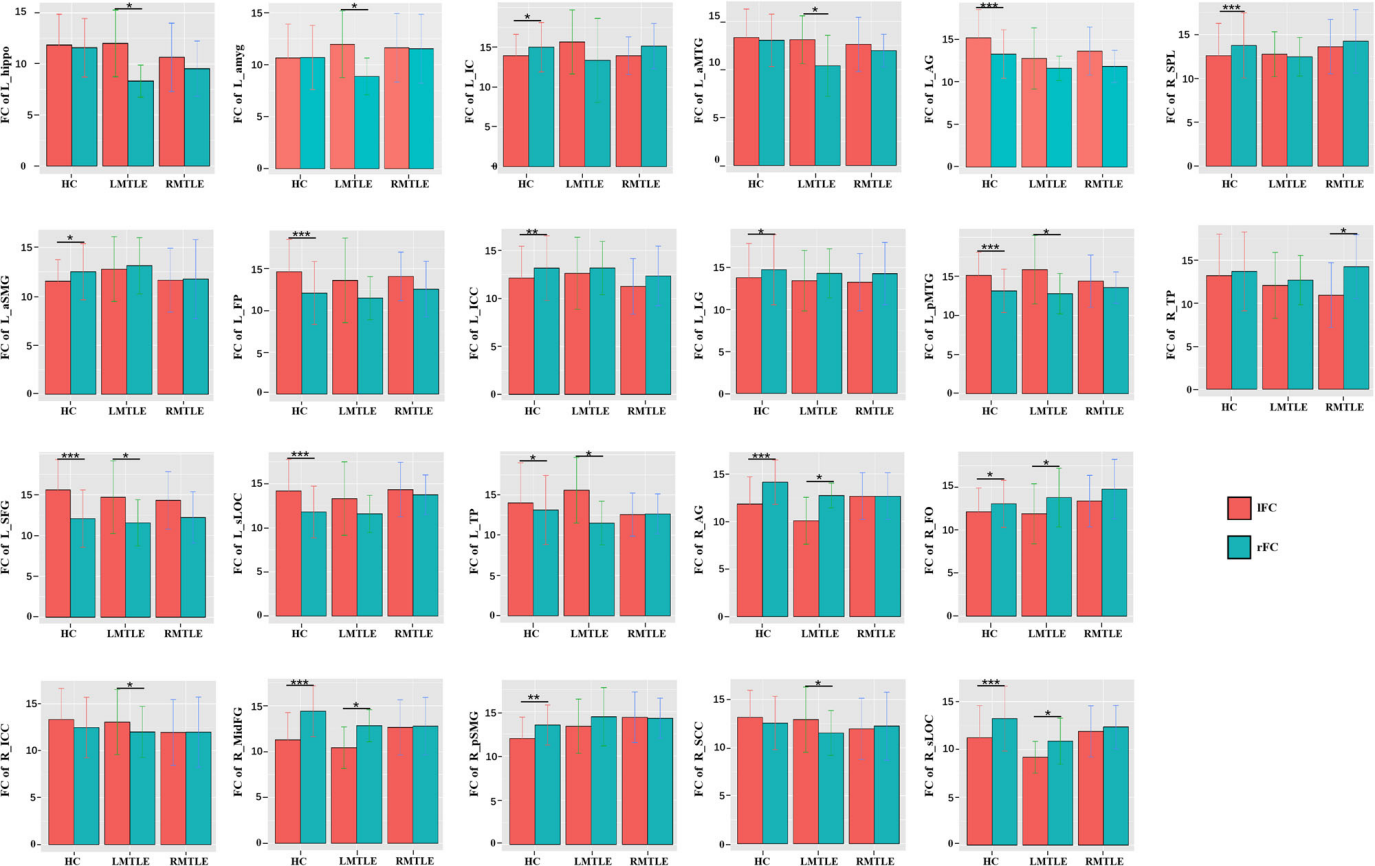
Visualization of the FC asymmetry of each ROI of the HC, LMTLE, and RMTLE groups (*P_FDR_*__* < 0.05; **P_FDR_*__* < 0.01; ***P_FDR_*__* < 0.001). Red represents left functional connectivity (lFC) and green represents right functional connectivity (rFC) of each ROI. The error bars represent standard deviation. L‐hippo, left hippocampus; L‐amyg, left amygdala; L‐IC, left insular cortex; L‐aMTG, anterior division of left middle temporal gyrus; L_AG, left angular gyrus; R_SPL, right superior parietal lobule; L_aSMG, anterior division of left supramarginal gyrus; L_FP, left frontal pole; L_ICC, left intracalcarine cortex; L_LG, left lingual gyrus; L_pMTG, posterior division of left middle temporal gyrus; R_TP, right temporal pole; L_SFG, left superior frontal gyrus; L_sLOC, superior division of left lateral occipital cortex; L_TP, left temporal pole; R_AG, right angular gyrus; R_FO, right frontal operculum cortex; R_ICC, right intracalcarine cortex; R_MidFG, right middle frontal gyrus; R_pSMG, posterior division of right supramarginal gyrus; R_SCC, right supracalcarine cortex; R_sLOC, superior division of right lateral occipital cortex

In the HC group, among the 37 ROIs (ROIs located in the midline of the brain were excluded in the asymmetric analysis), the FC of 16 ROIs showed asymmetric features, whereby significant differences were observed between rFC and lFC. In total, 10 ROIs showed rightward asymmetry (rFC > lFC), and 6 ROIs showed leftward asymmetry (lFC > rFC). In the LMTLE group, the number of ROIs that showed asymmetric features was reduced to 12 ROIs (rightward asymmetry/leftward asymmetry: 4/8). Only one ROI (R_TP) (rightward asymmetry/leftward asymmetry: 1/0) in RMTLE showed FC asymmetry after multiple correction. Specifically, some ROIs showed leftward asymmetry in the HC group were reduced in the patient groups, such as the left frontal pole (L_FP), L_AG, L_sLOC, while some rightward asymmetry (R_SPL, R_pSMG, L_aSMG, L_ICC, L_LG, and L_IC) were reduced as well. In addition, the FC of the L_hippo, L_amyg, L_aMTG, R_ICC, and R_SCC were symmetric in the HC group, whilst showing asymmetric characteristics in the LMTLE group (Figure 2).

### 3.4 Analysis of the functional connectivity asymmetry index

**FIGURE 3:**
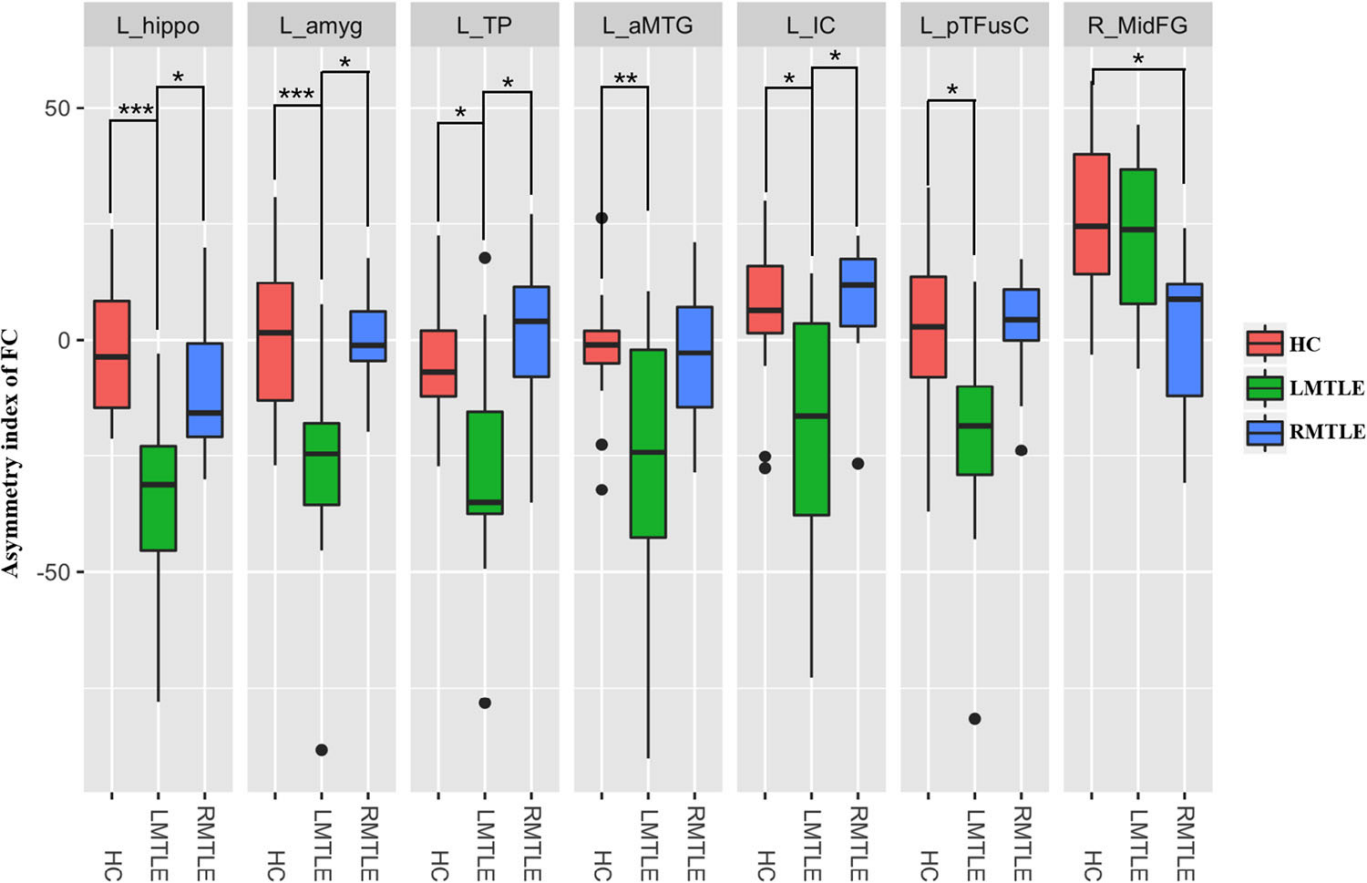
The asymmetry index (AI) of the FC differences of the ROIs among HC, LMTLE, and RMTLE. Red represents FC in HC; green, patients with LMTLE; blue, patients with RMTLE (*P_FDR_*__* < 0.05; **P_FDR_*__* < 0.01; ***P_FDR_*__* < 0.001). The black dots represent outliers. L‐hippo, left hippocampus; L‐amyg, left amygdala; L‐TP, left temporal pole; L‐aMTG, anterior division of left middle temporal gyrus; L‐IC, left insular cortex; L‐pTFusC, posterior division of left temporal fusiform cortex; R_MidFG, right middle frontal gyrus

The between‐group analysis of AIs showed that ROIs within left temporal lobe such as L_hippo (*P*_FDR_ = 0.000), L_amyg (*P*_FDR_ = 0.000), L_TP (*P*_FDR_ = 0.015), L_aMTG (*P*_FDR_ = 0.009), L_pTFusC (*P*_FDR_ = 0.025), and L_IC (*P*_FDR_ = 0.012) presented FC AI differences between HC and LMTLE, all of which presented reduced AI in LMTLE. R_MidFG (*P*_FDR_ = 0.037) showed reduced AI in RMTLE compared with HC. Four ROIs showed reduced AI in LMTLE compared with RMTLE, including ROIs in the left temporal lobe, that is, L‐hippo (*P*_FDR_ = 0.028), L‐amyg (*P*_FDR_ = 0.025), L_TP (*P*_FDR_ = 0.037), and ROIs involved the limbic system, that is, the L‐IC (*P*_FDR_ = 0.037) (Figure 3).

### 3.5 Correlation between abnormal functional connectivity and asymmetry and clinical variables of epilepsy

Pearson's correlation analyses were conducted to examine associations between the FC of significant abnormalities and AIs of significant lateralized ROIs and clinical features in LMTLE and RMTLE, respectively. However, no significant correlation was found between the FC and AI and the age of onset and epilepsy duration in both groups after multiple correction.

## 4 DISCUSSION

In this study, we analyzed the FC features and the functional asymmetry of the two hemispheres in patients with MTLE using rs‐fMRI. The areas located in the temporal lobe as well as extra‐temporal lobe showed abnormal FC and asymmetric features in MTLE. Interestingly, the FC changes and functional asymmetry alterations were distinctly affected in patients with LMTLE and RMTLE. RMTLE presented more extensive FC abnormalities than LMTLE. Regions in ipsilateral temporal lobe presented as central regions of abnormalities in both patient groups. Specifically, compared to controls, the increased FC revealed asymmetric features in LMTLE group, that is, involving most regions ipsilateral to the seizure focus. On the contrary, the reduced FC resulted to involve both hemispheres without obviously asymmetric features in LMTLE group. Similarly, both the reduced and increased FC revealed to involve both hemispheres in RMTLE group. In addition, our findings showed that the asymmetric characteristics of FC were reduced in MTLE compared with HC, with even more pronounced reduction for RMTLE group. Meanwhile, ROIs presented FC AI differences among the three groups were mostly involving left temporal lobe (L_hippo, L_amyg, L_TP, L_aMTG, and L_pTFusC).

Our study found that patients with MTLE had abnormal resting‐state FC that are not limited to the epileptogenic zone, but involving widespread regions of the brain compared with controls. Specifically, the ROIs in the ipsilateral temporal lobe, such as the hippocampus, amygdala, aMTG, TP, PaHC, and TFusC stood out as central regions of abnormal FC. The FC asymmetry of majority of these regions was also changed in patients with MTLE. It was expected because the epileptogenic zone is located in the temporal lobe and thus caused the most pronounced impairment within temporal lobe, as many previous studies have shown (Bettus et al., 2010; de Campos et al., 2016; Li et al., 2015; Pereira et al., 2010). Such FC abnormalities of MTLE also could be a biomarker of epileptogenic zone localization. Furthermore, both LMTLE and RMTLE groups showed decreased FC between bilateral temporal lobe and related areas. The reduced FC of interhemispheric connections might provide adaptive inhibition to protect contralateral areas in seizure propagation (Sirin et al., 2020). The increased FC between regions within ipsilateral temporal lobe and between regions in ipsilateral temporal lobe and bilateral IC, FO, and SPL possibly reflected compensatory mechanisms (Bettus et al., 2009). In addition, some ROIs located in the frontal lobe, parietal lobe, and occipital lobe also showed FC abnormalities and different asymmetric characteristics among the three groups. This supports the theory that MTLE is a network disease that could involve broad areas other than the temporal lobe (Bernhardt et al., 2011; Pittau et al., 2012).

The FC between the two hemispheres was asymmetric in the HC group presented in this study in congruence with several previous studies (Badzakova‐Trajkov et al., 2010; Toga & Thompson, 2003). Compared with HC, the asymmetric features of FC were reduced in both patient groups. For instance, the rightward asymmetry of the SMG presented in the HC group was reduced in the patient groups. The SMG was found to be involved in verbal working memory (Deschamps et al., 2014). Therefore, the reduced asymmetry of SMG in the patient groups may be associated with memory impairments and/or oroalimentary automatisms that are frequently observed in MTLE. Given that we did not test the relationship between these FC abnormalities and asymmetric features and cognitive performance, the clinical relevance of these findings can only be speculative. Nevertheless, the hemispheric asymmetry of certain functions such as language, spatial attention, and memory in the human brain was thought to be beneficial for functioning (Badzakova‐Trajkov et al., 2010; Habib et al., 2003; Toga & Thompson, 2003). It is also known that distinct functions and a division of labor between the left and right hemispheres may improve overall cognitive ability and performance (Gotts et al., 2013). Thus, the reduced FC asymmetry in patients with MTLE might play an important role in cognitive impairment.

Interestingly, the FC changes and functional asymmetry alterations appeared differently between LMTLE and RMTLE in this study. Compared to controls, RMTLE presented more extensive FC abnormalities and asymmetric characteristics than LMTLE. Specifically, the increased FC revealed asymmetric features in LMTLE group, that is, involving most regions in left hemisphere. The functional asymmetry differences between LMTLE and RMTLE were mostly involving left temporal lobe (L_TP, L_Hippo, and L_Amyg) as well. The findings about LMTLE and RMTLE group differences in FC were not completely consistent. Some studies showed that LMTLE appeared more extensive network impairment than RMTLE (Haneef et al., 2014; Pereira et al., 2010) while others showed the opposite results (Steiger et al., 2017; Zhang et al., 2010). These discrepancies among the studies probably reflected variations in methodology. For example, both Pereira and Haneef defined bilateral hippocampus as seeds and detected more pronounced FC changes in LMTLE than RMTLE. However, when considered a more widespread changes and involved more regions outside temporal lobe, the results would be different. For instance, according to Steiger's report, RMTLE showed more pronounced functional networks disruption encompassed bilateral amygdala, limbic, cortical, subcortical, and brainstem regions during the perception of fearful faces. Also, Zhang et al. reported that RMTLE patients showed more extensive FC changes of the default‐mode network than LMTLE. These findings were congruent with our study. Another reason of the discrepancies between LMTLE and RMTLE probably reflected the different adaptive and compensatory mechanisms of cognitive impairments (Ives‐Deliperi & Butler, 2021). The relatively small sample size could also contribute to the discrepancies among these studies and thus multi‐center studies that enrolled large samples of patients were necessary. Anyhow, these findings supported the theory that LMTLE and RMTLE are etiologically distinct and pathologically different syndromes from the outset (Zhao et al., 2019).

Bilateral insular cortex presented reduced FC with ipsilateral hippocampus both in LMTLE and RMTLE. Functional AI of regions involved left limbic system as left hippocampus, left amygdala, and left insula also presented differences among HC, LMTLE, and RMTLE. In addition, compared with LMTLE, more regional connections were reduced in RMTLE, most predominantly involving reduced FC between ipsilateral IC and ipsilateral PaHC, amygdala, and TP. It was congruent with the previous study that showed more pronounced reductions of limbic FC in right TLE (Chiang et al., 2014). The limbic system is a complex neural circuit predominantly involved in memory and emotional output and has repeatedly been shown to affect TLE (Chan et al., 1997; Liacu et al., 2012; Zhao et al., 2019). The majority of studies showed bilateral limbic system impairment in TLE (Concha et al., 2005; Liacu et al., 2012), but few analyzed the asymmetric alterations of the limbic system. In our study, the functional AI of left limbic system was different between LMTLE and RMTLE, mostly due to the reduced rFC of these regions in LMTLE. It probably reflected the reduced compensation of left limbic system in LMTLE, because it was reported that the left hemisphere has a greater preference for within‐hemisphere interactions and interacts less with the right hemisphere (Gotts et al., 2013; Li et al., 2015). Thus, when impairment involved the left limbic system, the compensatory pathway could not be as effective as the right limbic system. Taken together, these data provide evidence that the bilateral limbic systems play different roles in the communication and compensatory mechanisms associated with the bilateral sides of the brain.

There are some limitations to this study. First, the patient population was relatively small. Therefore, the findings should be interpreted with caution and future studies with more patients are required. Second, due to the lack of cognitive evaluations, the clinical relevance of these findings is only speculative. However, the previous study found that some cognitive functions such as language are associated with brain asymmetry (Sarica et al., 2018; Toga & Thompson, 2003). We would include cognitive evaluations in our future study. Third, we did not conduct fine screening of epilepsy comorbidities of the patients. Some conditions comorbid with epilepsy, such as autism, are known to be associated with functional asymmetry. We should record details about epilepsy comorbidities in the future.

## 5 CONCLUSION

In conclusion, the abnormal FC and asymmetric features of MTLE are altered and involve both the temporal lobe and extra‐temporal lobe. Furthermore, the altered FC and asymmetric features were distinctly affected in LMTLE and RMTLE.

## CONFLICT OF INTEREST

The authors declare that they have no conflict of interest.

### PEER REVIEW

The peer review history for this article is available at https://publons.com/publon/10.1002/brb3.2484

## Supporting information



Supporting information

## Data Availability

The data that support the findings of this study are available from the corresponding author upon reasonable request.
