# Supplementary material for: Interhemispheric functional connectivity asymmetry is distinctly affected in left and right mesial temporal lobe epilepsy
Source: Brain Behav. 2022 Feb 14;12(3):e2484. doi: 10.1002/brb3.2484 (PMC8933759; doi:10.1002/brb3.2484)
Supplement: Supplementary file 1 — Supporting information [file BRB3-12-e2484-s001.docx]

Supplementary Material

**Interhemispheric functional connectivity asymmetry is distinctly affected in left and right mesial temporal lobe epilepsy**

**Xu Zhao ^1^, Huicong kang ^2^, Zhiqiang Zhou ^3^, Ying Hu ^1^, Juan Li ^1^, Shihui Li ^1^, Jia Li ^1^, Wenzhen Zhu ^1*^**

^1^Department of Radiology, Tongji Hospital, Tongji Medical College, Huazhong University of Science and Technology, Wuhan, China

^2^Department of Neurology, Tongji Hospital, Tongji Medical College, Huazhong University of Science and Technology, Wuhan, China

^3^Department of Anesthesiology and Pain Medicine, Tongji Hospital, Tongji Medical College, Huazhong University of Science and Technology, Wuhan, China

***Correspondence:**

Wen-zhen Zhu

zhuwenzhen8612@163.com

**Supplementary Tables**

**Table 1. Demographic and clinical information for MTLE patients**

| **NO.** | **Groups** | **Gender** | **Age (years)** | **Age at onset (years)** | **Duration** | **Drug trials** |
| --- | --- | --- | --- | --- | --- | --- |
| 1 | LMTLE | M | 26 | 16 | 10 | VPA, LTG |
| 2 | LMTLE | F | 21 | 16 | 5 | OXC |
| 3 | LMTLE | M | 19 | 2 | 17 | OXC |
| 4 | LMTLE | M | 27 | 7 | 20 | VPA, LTG |
| 5 | LMTLE | F | 50 | 30 | 20 | OXC, VPA, LTG |
| 6 | LMTLE | M | 32 | 29 | 3 | TPX, CBZ |
| 7 | LMTLE | M | 46 | 20 | 26 | VPA, LTG, CLZ |
| 8 | LMTLE | M | 18 | 17 | 0.92 | CM |
| 9 | LMTLE | M | 27 | 25 | 2 | CM |
| 10 | LMTLE | M | 28 | 8 | 20 | OXC, VPA |
| 11 | LMTLE | F | 33 | 13 | 20 | VPA, CBZ |
| 12 | LMTLE | M | 24 | 14 | 10 | VPA |
| 13 | RMTLE | M | 25 | 13 | 12 | CM |
| 14 | RMTLE | M | 28 | 12 | 16 | LTG, CBZ |
| 15 | RMTLE | F | 49 | 24 | 25 | VPA, CBZ |
| 16 | RMTLE | F | 36 | 30 | 6 | TPX |
| 17 | RMTLE | F | 22 | 11 | 11 | OXC, LTG, TPX |
| 18 | RMTLE | M | 23 | 13 | 10 | OXC |
| 19 | RMTLE | M | 48 | 28 | 20 | OXC, VPA, CLZ |
| 20 | RMTLE | M | 22 | 4 | 18 | CM |
| 21 | RMTLE | F | 32 | 25 | 7 | CBZ, TPX |
| 22 | RMTLE | F | 41 | 29 | 12 | LEV, OXC |
| 23 | RMTLE | M | 24 | 21 | 3 | VPA, CM |

LMTLE, left mesial temporal lobe epilepsy with hippocampal sclerosis; RMTLE, right mesial temporal lobe epilepsy with hippocampal sclerosis. F, female; M, male. VPA, valproic acid; LTG, lamotrigine; OXC, oxcarbazepine; TPX, topiramate; CBZ, carbamazepine; CLZ, clonazepam; CM, Chinese medicine; LEV, levetiracetam.

**Table 2.** **ROI-to-ROI functional connectivity differences between LMTLE and control**

| Analysis Unit | *T* | *Puncorrected* | *P_FDR_* |
| --- | --- | --- | --- |
| Reduced FC in LMTLE than control | | | |
| Hippocampus l-Hippocampus r | -6.56 | 0 | 0 |
| Hippocampus l-LG l | -4.3 | 0.0001 | 0.0009 |
| Hippocampus l-aMTG r | -4.22 | 0.0001 | 0.0009 |
| Hippocampus l-PC | -4.21 | 0.0001 | 0.0009 |
| Hippocampus l-SCC l | -4.05 | 0.0001 | 0.0011 |
| Hippocampus l-pPaHC r | -3.64 | 0.0005 | 0.0029 |
| Hippocampus l-ICC r | -3.57 | 0.0006 | 0.003 |
| Hippocampus l-LG r | -3.49 | 0.0007 | 0.0033 |
| Hippocampus l-SFG l | -3.37 | 0.001 | 0.0037 |
| Hippocampus l-ICC l | -3.35 | 0.001 | 0.0037 |
| Hippocampus l-Precuneous | -3.33 | 0.0011 | 0.0037 |
| Hippocampus l-aPaHC r | -3.18 | 0.0016 | 0.0051 |
| Hippocampus l-sLOC r | -3.12 | 0.0019 | 0.0055 |
| Hippocampus l-SCC r | -3.09 | 0.002 | 0.0055 |
| Hippocampus l-sLOC l | -2.74 | 0.0049 | 0.0125 |
| Hippocampus l-MedFC | -2.23 | 0.0163 | 0.0375 |
| Hippocampus l-Amygdala r | -2.22 | 0.0168 | 0.0375 |
| Hippocampus l-AG l | -2.11 | 0.0213 | 0.045 |
| aMTG l -aMTG r | -5.37 | 0 | 0.0001 |
| aMTG l -SFG l | -3.4 | 0.0009 | 0.0167 |
| aMTG l -aPaHC r | -3.04 | 0.0023 | 0.0294 |
| aMTG l -AG l | -2.9 | 0.0033 | 0.0309 |
| TP l -aMTG r | -4.1 | 0.0001 | 0.0048 |
| TP l -TP r | -3.29 | 0.0012 | 0.0206 |
| TP l -AG l | -3.17 | 0.0016 | 0.0206 |
| Amygdala l-Amygdala r | -4.81 | 0 | 0.0006 |
| LG l -pPaHC l | -3.15 | 0.0017 | 0.0326 |
| aPaHC r -sLOC r | -3.11 | 0.0019 | 0.0294 |
|  | | | |
| Increased FC in LMTLE than control | | | |
| Hippocampus l-IC l | 5 | 0 | 0.0003 |
| Hippocampus l-IC r | 3.21 | 0.0015 | 0.0113 |
| Hippocampus l-FO l | 3.25 | 0.0013 | 0.0113 |
| Hippocampus l-FO r | 3.89 | 0.0002 | 0.0041 |
| Hippocampus l-Amygdala l | 3.77 | 0.0003 | 0.0041 |
| Hippocampus l-aSMG l | 2.9 | 0.0033 | 0.0207 |
| IC l -Amygdala l | 3.88 | 0.0002 | 0.0045 |
| IC l -pPaHC l | 3.18 | 0.0016 | 0.0204 |
| IC l -TP l | 2.84 | 0.0038 | 0.0366 |
| pTFusC l -aMTG l | 3.29 | 0.0012 | 0.0228 |
| pTFusC l -TP l | 3.81 | 0.0003 | 0.0109 |
| IC r -Hippocampus r | 3.01 | 0.0025 | 0.0472 |

Abbreviations: AG, angular gyrus; aMTG, anterior division of middle temporal gyrus; aPaHC, anterior division of parahippocampal gyrus; aSMG, anterior division of supramarginal gyrus; FDR, false discovery rate; FO, frontal operculum cortex; IC, insular cortex; ICC, intracalcarine cortex; LG, lingual gyrus; LMTLE, left mesial temporal lobe epilepsy with hippocampal sclerosis; MedFC, frontal medial cortex, PC, posterior division of cingulate gyrus; pPaHC, posterior division of parahippocampal gyrus; pTFusC, posterior division of temporal fusiform cortex; RMTLE, right mesial temporal lobe epilepsy with hippocampal sclerosis; ROI, region of interest; SCC, supracalcarine cortex; SFG, superior frontal gyrus; sLOC, superior division of lateral occipital cortex; TP, temporal pole.

**Table 3. ROI-to-ROI functional connectivity differences between RMTLE and control**

| Analysis Unit | *T* | *Puncorrected* | *P_FDR_* |
| --- | --- | --- | --- |
| Reduced FC in RMTLE than control | | | |
| Hippocampus r-Hippocampus l | -5.76 | 0 | 0 |
| Hippocampus r-PC | -5.39 | 0 | 0.0001 |
| Hippocampus r-aMTG r | -4.85 | 0 | 0.0002 |
| Hippocampus r-sLOC l | -4.76 | 0 | 0.0002 |
| Hippocampus r-AG l | -4.35 | 0.0001 | 0.0005 |
| Hippocampus r-pPaHC l | -4.27 | 0.0001 | 0.0005 |
| Hippocampus r-Precuneous | -4.26 | 0.0001 | 0.0005 |
| Hippocampus r-aMTG l | -4.17 | 0.0001 | 0.0005 |
| Hippocampus r-pMTG l | -3.55 | 0.0006 | 0.0026 |
| Hippocampus r-MedFC | -3.36 | 0.001 | 0.0039 |
| Hippocampus r-sLOC r | -3.25 | 0.0013 | 0.0046 |
| Hippocampus r-SFG l | -3.17 | 0.0017 | 0.0054 |
| Hippocampus r-AG r | -2.63 | 0.0066 | 0.0178 |
| Hippocampus r-pTFusC l | -2.52 | 0.0085 | 0.0216 |
| Hippocampus r-SCC l | -2.75 | 0.0049 | 0.0142 |
| Hippocampus r-MidFG r | -2.37 | 0.0119 | 0.0271 |
| Hippocampus r-FP l | -2.37 | 0.0121 | 0.0271 |
| Amygdala r-AG r | -3.79 | 0.0003 | 0.0055 |
| Amygdala r-PC | -3.71 | 0.0004 | 0.0055 |
| Amygdala r-MidFG r | -3.68 | 0.0004 | 0.0055 |
| Amygdala r-Amygdala l | -3.52 | 0.0007 | 0.0062 |
| Amygdala r-AG l | -3.41 | 0.0009 | 0.0067 |
| Amygdala r-Precuneous | -3.09 | 0.002 | 0.013 |
| Amygdala r-sLOC l | -2.93 | 0.0031 | 0.0171 |
| Amygdala r-sLOC r | -2.68 | 0.0057 | 0.0246 |
| Amygdala r-pMTG l | -2.68 | 0.0058 | 0.0246 |
| aMTG r -aMTG l | -3.56 | 0.0006 | 0.006 |
| aMTG r -AG l | -3.54 | 0.0006 | 0.006 |
| aMTG r -Amygdala l | -2.79 | 0.0044 | 0.0301 |
| aMTG r -pPaHC l | -2.63 | 0.0065 | 0.0301 |
| aMTG r -pMTG l | -2.6 | 0.0069 | 0.0301 |
| aMTG r -SFG l | -2.6 | 0.0071 | 0.0301 |
| aMTG r -TP l | -2.59 | 0.0071 | 0.0301 |
| TP r -AG l | -4.01 | 0.0002 | 0.0041 |
| TP r -aMTG l | -3.78 | 0.0003 | 0.0041 |
| TP r -pMTG l | -3.78 | 0.0003 | 0.0041 |
| TP r -PC | -3.37 | 0.001 | 0.0094 |
| TP r -Precuneous | -2.91 | 0.0033 | 0.0242 |
| TP r -TP l | -2.85 | 0.0038 | 0.0242 |
| Hippocampus l-aMTG r | -3.72 | 0.0004 | 0.0073 |
| Hippocampus l-SFG l | -2.99 | 0.0027 | 0.0339 |
| Hippocampus l-pPaHC r | -2.77 | 0.0046 | 0.0434 |
| Hippocampus l-aPaHC r | -2.68 | 0.0057 | 0.0434 |
| PC -aPaHC r | -3.24 | 0.0014 | 0.0133 |
| TP l -AG l | -3.48 | 0.0007 | 0.0277 |
| TP l -pMTG l | -2.89 | 0.0035 | 0.0484 |
| Precuneous -IC l | -2.92 | 0.0032 | 0.0312 |
| sLOC l -SPL l | -2.95 | 0.0029 | 0.0386 |
| sLOC l -aPaHC r | -2.82 | 0.0041 | 0.0386 |
| AG l -aMTG l | -2.58 | 0.0074 | 0.0444 |
| AG l -Hippocampus l | -2.53 | 0.0082 | 0.0444 |
|  | | | |
| Increased FC in RMTLE than control | | | |
| Hippocampus r-IC r | 5.01 | 0 | 0.0002 |
| Hippocampus r-FO r | 4.95 | 0 | 0.0002 |
| Hippocampus r-SPL l | 4.17 | 0.0001 | 0.0014 |
| Hippocampus r-pSMG r | 4 | 0.0002 | 0.0017 |
| Hippocampus r-aSMG r | 3.9 | 0.0002 | 0.0018 |
| Hippocampus r-SPL r | 3.71 | 0.0004 | 0.0025 |
| Hippocampus r-IC l | 3.32 | 0.0011 | 0.0061 |
| Hippocampus r-FO l | 3.08 | 0.0021 | 0.0102 |
| Hippocampus r-aSMG l | 2.66 | 0.0061 | 0.0258 |
| pPaHC r -FO l | 4.04 | 0.0002 | 0.0032 |
| pPaHC r -IC r | 3.94 | 0.0002 | 0.0032 |
| pPaHC r -IC l | 3 | 0.0026 | 0.0197 |
| pPaHC r -FO r | 3.87 | 0.0002 | 0.0032 |
| pPaHC r -pSMG r | 3 | 0.0026 | 0.0197 |
| aPaHC r -FO r | 3.91 | 0.0002 | 0.0054 |
| aPaHC r -IC r | 3.82 | 0.0003 | 0.0054 |
| aPaHC r -FO l | 3.24 | 0.0014 | 0.0178 |
| aPaHC r -pSMG r | 2.8 | 0.0043 | 0.0406 |
| aPaHC r -IC l | 2.71 | 0.0054 | 0.0408 |
| pSMG r -Amygdala l | 3.18 | 0.0017 | 0.0179 |
| pSMG r -pPaHC l | 2.98 | 0.0028 | 0.0179 |
| pSMG r -Hippocampus l | 2.97 | 0.0028 | 0.0179 |
| pSMG r -aPaHC r | 2.8 | 0.0043 | 0.0232 |
| pSMG r -aTFusC l | 5.02 | 0 | 0.0004 |
| IC r -TP r | 4.47 | 0 | 0.0009 |
| IC r -pPaHC r | 3.94 | 0.0002 | 0.0026 |
| IC r -aPaHC r | 3.82 | 0.0003 | 0.0027 |
| IC r -Amygdala r | 2.89 | 0.0034 | 0.0261 |
| FO r -pPaHC l | 3.27 | 0.0013 | 0.0097 |
| FO r -LG l | 2.77 | 0.0046 | 0.0249 |
| FO r -TP r | 2.71 | 0.0053 | 0.0252 |
| FO r -LG r | 2.44 | 0.0103 | 0.0435 |
| MidFG r -SFG l | 4.69 | 0 | 0.0009 |
| MidFG r -FP l | 3.87 | 0.0003 | 0.0048 |
| MidFG r -aTFusC l | 2.85 | 0.0038 | 0.0475 |
| sLOC l -PC | 3.98 | 0.0002 | 0.0039 |
| sLOC l -Precuneous | 3.94 | 0.0002 | 0.0039 |
| Amygdala l-FO r | 3.19 | 0.0016 | 0.0239 |
| Amygdala l-SPL r | 3.12 | 0.0019 | 0.0239 |
| Hippocampus l-FO r | 3.54 | 0.0006 | 0.0234 |

Abbreviations: AG, angular gyrus; aMTG, anterior division of middle temporal gyrus; aPaHC, anterior division of parahippocampal gyrus; aSMG, anterior division of supramarginal gyrus; aTFusC, anterior division of temporal fusiform cortex; FDR, false discovery rate; FO, frontal operculum cortex; FP, frontal pole; IC, insular cortex; LMTLE, left mesial temporal lobe epilepsy with hippocampal sclerosis; LG, lingual gyrus; MedFC, frontal medial cortex; MidFG, middle frontal gyrus; PC, posterior division of cingulate gyrus; pMTG, posterior division of middle temporal gyrus; pPaHC, posterior division of parahippocampal gyrus; pSMG, posterior division of supramarginal gyrus; pTFusC, posterior division of temporal fusiform cortex; RMTLE, right mesial temporal lobe epilepsy with hippocampal sclerosis; ROI, region of interest; SCC, supracalcarine cortex; SFG, superior frontal gyrus; SPL, superior parietal lobule; sLOC, superior division of lateral occipital cortex; TP, temporal pole.

**Table 4. ROI-to-ROI functional connectivity differences between LMTLE and RMTLE**

| Analysis Unit | *T* | *Puncorrected* | *P_FDR_* |
| --- | --- | --- | --- |
| Reduced FC in LMTLE than RMTLE | | | |
| aTFusC l -pSMG r | -4.85 | 0 | 0.0016 |
| aTFusC l -ICC l | -3.7 | 0.0007 | 0.0126 |
| aTFusC l -MidFG r | -2.78 | 0.0056 | 0.0425 |
| aTFusC l -SCC r | -2.63 | 0.0077 | 0.049 |
| aTFusC l -LG l | -3.16 | 0.0024 | 0.0301 |
| aTFusC l -aSMG r | -3.01 | 0.0033 | 0.0313 |
| IC r -TP r | -3.86 | 0.0005 | 0.0174 |
| LG l -Amygdala l | -3.01 | 0.0033 | 0.0419 |
| LG l -Hippocampus l | -3.58 | 0.0009 | 0.0334 |
| sLOC l -PC | -3.51 | 0.001 | 0.0394 |
|  | | | |
| Increased FC in LMTLE than RMTLE | | | |
| Amygdala r-AG l | 4.51 | 0.0001 | 0.0037 |
| Amygdala r-sLOC l | 3.09 | 0.0028 | 0.0303 |
| Amygdala r-pMTG l | 3.03 | 0.0032 | 0.0303 |
| Amygdala r-PC | 3.5 | 0.0011 | 0.0203 |
| Hippocampus r-sLOC l | 3.82 | 0.0005 | 0.019 |
| Hippocampus r-PC | 3.53 | 0.001 | 0.019 |
| Hippocampus r-Precuneous | 3.18 | 0.0022 | 0.0283 |
| SCC l -SCC r | 3.9 | 0.0004 | 0.0148 |
| SCC l -ICC r | 3.63 | 0.0008 | 0.0148 |
| pTFusC l -TP l | 3.74 | 0.0006 | 0.023 |

Abbreviations: AG, angular gyrus; aSMG, anterior division of supramarginal gyrus; aTFusC, anterior division of temporal fusiform cortex; FDR, false discovery rate; IC, insular cortex; ICC, intracalcarine cortex; LG, lingual gyrus; LMTLE, left mesial temporal lobe epilepsy with hippocampal sclerosis; MidFG, middle frontal gyrus; PC, posterior division of cingulate gyrus; pMTG, posterior division of middle temporal gyrus; pSMG, posterior division of supramarginal gyrus; pTFusC, posterior division of temporal fusiform cortex; RMTLE, right mesial temporal lobe epilepsy with hippocampal sclerosis; ROI, region of interest; SCC, supracalcarine cortex; sLOC, superior division of lateral occipital cortex; TP, temporal pole.
